# Optimizing Titanium Carbide-Silver Oxide Nanostructures for Targeted Cancer Therapy: Synthesis, Functionalization, and In Vitro Evaluations

**DOI:** 10.7759/cureus.69757

**Published:** 2024-09-19

**Authors:** Dharshini B., Geetha A., Vasugi S., Balachandran S., Ilangovar I. G. K.

**Affiliations:** 1 Department of Physiology, Saveetha Dental College and Hospitals, Saveetha Institute of Medical and Technical Sciences, Saveetha University, Chennai, IND

**Keywords:** anticancer activity, colorimetric assay, in vitro evaluation, mxene, silver oxide

## Abstract

Introduction

Cancer remains a significant health challenge, and nanoparticles (NPs) are promising candidates for cancer treatment due to their unique physicochemical properties and ability to selectively target tumour cells. Two-dimensional (2D) nanomaterials, such as MXenes, have attracted interest due to their electronic structures, optical properties, catalytic abilities, and exceptional physicochemical attributes. MXenes are highly suitable for surface functionalization or modification, and their unique properties make them promising candidates for various applications in the biological field. Silver-based compounds have shown remarkable potential in biomedical fields, with silver oxide (Ag₂O) NPs finding applications in various domains. The fabrication of titanium carbide (Ti₃C₂)-Ag₂O heterostructures has been investigated for their anti-cancer properties by conducting cell viability assays on different cell lines.

Aim

To synthesize and characterize Ti₃C₂-Ag₂O, and to assess its in vitro anti-cancer activity.

Materials and methods

Ti₃C₂ synthesis begins by dissolving Ti₃AlC₂ powder in a 50% v/v hydrofluoric (HF) acid solution, allowing the aluminium to be etched away. This process should be conducted with continuous stirring for 24 to 48 hours at ambient temperature. Following this, filter the resulting suspension to eliminate aluminium particles and HF, and subsequently wash the Ti₃C₂ MXene with distilled water until a neutral pH is attained. The MXene should then be dispersed in ethanol, and sonication in deionized (DI) water or an alternative solvent should be employed to achieve exfoliation into monolayer or few-layer MXenes. To prepare Ag₂O NPs, dissolve silver nitrate (AgNO₃) in DI water to create a 0.1 M solution, and concurrently prepare a separate 0.1 M sodium hydroxide (NaOH) solution. Introduce the NaOH solution to the AgNO₃ while stirring until a precipitate is observed. The mixture should then be filtered, washed with distilled water, and the NPs dried at 60°C for 12 hours. To fabricate the MXene-Ag₂O composite, disperse the MXene nanoflakes in a solvent through sonication, incorporate the Ag₂O NPs, and stir the mixture for 24 hours. Finally, centrifuge the resultant mixture to isolate the composite, wash it with solvent, and dry it under vacuum conditions.

Results

The presence of Ag₂O particles on Ti₃C₂ nanosheets was observed, and the high crystallinity of the compound was confirmed through X-ray diffraction (XRD), energy-dispersive spectroscopy (EDS), and scanning electron microscopy (SEM) analyses. These tests verified that the compound was free of impurities and exhibited anti-cancer properties.

Conclusion

The synthesis of Ti₃C₂ MXenes and Ag₂O NPs was achieved and confirmed through structural characterization methods, including SEM, XRD, and EDS. SEM provided detailed insights into the morphology and distribution of the nanostructures, while XRD and EDS verified their phase purity and elemental composition. Functionalization strategies were employed to enhance the stability and bioactivity of the nanocomposites. In vitro evaluations demonstrated promising anti-cancer activity, indicating that the Ti₃C₂-Ag₂O composites effectively target and inhibit cancer cell growth.

## Introduction

Cancer continues to pose a significant health challenge, with its incidence on the rise. Despite the progress made in cancer treatment, there remains a pressing need for more effective and targeted therapies, as traditional systemic drug delivery often leads to off-target toxicity and swift drug clearance [1]. The unique physicochemical properties of nanoparticles (NPs), combined with their ability to selectively target tumour cells, make them promising candidates for cancer treatment. NPs can be engineered to deliver drugs, genes, or imaging agents directly to tumour cells, thereby reducing toxicity to surrounding healthy tissues [2]. Their size, shape, and surface characteristics can be precisely adjusted to improve therapeutic efficacy and minimize adverse side effects. Additionally, NPs have the potential to overcome biological barriers that limit the effectiveness of traditional cancer therapies, such as poor solubility, rapid clearance, and restricted penetration into solid tumours [3]. Two-dimensional (2D) nanomaterials have attracted considerable interest recently due to their distinctive electronic structures, optical properties, catalytic abilities, and exceptional physicochemical attributes. These materials consist of single-atomic or polyatomic layers, which can be either elemental heteromorphs or composites and are connected by strong transverse covalent bonds and van der Waals interactions. Given the rapid spread of cancer cells throughout the body, researchers are focusing on developing more accurate and effective diagnostic methods and therapeutic strategies to combat tumours [4].

MXenes, with the general formula Mn+1XnTx and a range of functional groups such as -O, -OH, -Cl, and -F, are highly suitable for surface functionalization or modification. This versatility enables the creation of advanced micro and nanosystems with multifunctional properties. Various approaches for surface functionalization of MXenes have been explored, utilizing both covalent and noncovalent modification techniques [5]. The surface terminations of MXenes, including -O, -F, and -OH, combined with their transition metal carbide and nitride core, impart a unique combination of enhanced hydrophilicity and metallic conductivity. MXenes are distinguished by their exceptional physicochemical properties, such as high electrical conductivity, large surface areas, and significant optical, magnetic, thermal, and mechanical characteristics. These attributes make MXenes a promising candidate for a wide range of future applications in the biological field [6]. The inherent flexibility of MXenes, combined with their 2D structure and layered configuration, promotes effective interactions with various materials. Furthermore, MXenes are characterized by biocompatibility and biodegradability, facilitating their efficient removal from the human body. These qualities contribute to significant advancements in nanomedicine, particularly in cancer treatment, bioimaging, biosensing, and antibacterial applications [7]. Combining MXenes with graphene in hybrid or composite forms can fulfil numerous unmet requirements across diverse sectors, especially in medicine and biomedical engineering. MXenes hold substantial potential for effective and minimally invasive cancer therapies, leveraging their unique photothermal, chemotherapeutic, and photodynamic properties. Researchers have investigated their capabilities for the targeted delivery of anticancer drugs and medicines, employing heat and light as treatment modalities. The antitumor efficacy of the graphene platform has been further improved through chemical functionalization, while graphene-based combination therapies of chemotherapy and phototherapy enable precise targeting of cancer cells [8]. The utilization of nanomaterials is increasingly recognized for their diverse applications in the medical, environmental, and electrical sectors. Notably, silver-based compounds have demonstrated remarkable potential in biomedical fields, exhibiting properties such as antimicrobial, anticancer, antioxidant, anticoagulant, thrombolytic, and larvicidal activities. Significant research efforts have been directed towards the synthesis of silver NPs, particularly for their application in wound healing and prosthetic devices, as well as their incorporation into textiles and clothing. Silver reacts with oxygen to produce silver oxide (Ag₂O), and Ag₂O NPs find applications in various domains, including photovoltaic devices, plasmonic photonic devices, optical storage solutions, and cathodes in batteries. Additionally, Ag₂O NPs are recognized for their potent antimicrobial properties. These NPs can be synthesized through electrochemical or thermochemical methods, as well as reactive sputtering techniques. However, these processes often necessitate specialized equipment and the use of various toxic chemicals [9].

A p-type semiconductor, Ag₂O, exhibits a band gap of 1.46 eV. It has found extensive application as a catalyst in the synthesis of ethylene oxide and serves as a co-catalyst in palladium-catalysed organic reactions. A straightforward method for colourimetric detection of sulphite was the observation that the oxidase-like activity of Ag₂O NPs is inhibited by sulphite. When it comes to the photocatalytic degradation of methylene blue under visible light, Ag₂O demonstrates superior efficiency compared to silver colloid and various other metal oxides. The diverse crystal structures of Ag₂O contribute to a range of intriguing physicochemical properties, including catalytic, electrochemical, electronic, and optical characteristics [10]. The study focused on the fabrication of titanium carbide (Ti₃C₂)-Ag₂O heterostructures by a modified method, including hydrofluoric (HF) acid etching followed by hydrothermal processing. Ti₃C₂-Ag₂O materials exhibited unique properties during characterization. The presence of Ti₃C₂-Ag₂O nanomaterials was confirmed by X-ray diffraction (XRD), scanning electron microscopy (SEM), and energy-dispersive spectroscopy (EDS), which provided insights into their crystalline structure, phase composition, surface morphology, and elemental composition [11]. In addition, the study investigated the anti-cancer properties by conducting cell viability assays on different cell lines to evaluate the biocompatibility of the Ti₃C₂-Ag₂O formulation.

## Materials and methods

Synthesis of Ti₃C₂

The synthesis of MXene (Ti₃C₂) was carried out using commercially sourced Ti₃AlC₂ MAX phase as the precursor material. A 2.5 g sample of Ti₃AlC₂ black powder was mixed with 60 mL of 40% HF acid for 24 hours. During the etching process, a magnetic stirring mechanism was employed to ensure consistent heating at 40°C and continuous agitation, thereby enhancing the efficiency of the reaction over 24 hours. Approximately 10 cm³ of HF was utilized for every gram of the initial material, with small quantities of Ti₃AlC₂ being added to the HF solution throughout the reaction. This process resulted in a highly exothermic reaction and considerable hydrogen gas evolution. The resulting mixture served as an etchant, effectively targeting the aluminium layers within Ti₃AlC₂ while safeguarding the desired MXene layers. To neutralize the acidity of the compound, the material underwent extensive washing until a pH of 8 was achieved.

Synthesis of Ag₂O nanocomposite

To synthesize Ag₂O NPs, we initiated the process by dissolving 0.17 g of silver nitrate (AgNO₃) in 100 mL of deionized (DI) water to form an AgNO₃ solution. We gradually added sodium hydroxide (NaOH) or ammonium hydroxide (NH₄OH) to this solution while continuously stirring. The introduction of the base resulted in a colour change, indicating the formation of Ag₂O NPs. We continued to stir the mixture for one to two hours to ensure the complete formation of the Ag₂O NPs. Upon completion of the reaction, we centrifuged the mixture to isolate the Ag₂O NPs from the solution. We rinsed the collected NPs with DI water to eliminate residual ions or reactants. Finally, we redispered the purified Ag₂O NPs in DI water, preparing them for subsequent composite preparation.

Preparation of Ti₃C₂-Ag₂O NP composite

To create the Ti₃C₂-Ag₂O NP composite, initiate the process by combining a suspension of Ti₃C₂ with a suspension of Ag₂O NPs. The proportion of Ti₃C₂ to Ag₂O can be adjusted according to the specific properties desired in the final composite. Stir the mixture at ambient temperature for several hours, generally between two and six hours, to promote the attachment of Ag₂O NPs to the Ti₃C₂ nanosheets. To improve stability, it may be beneficial to incorporate a surfactant, such as polyethylene glycol (PEG), to stabilize the composite, or to conduct a chemical reduction process to further secure the NPs onto the MXene surface. Following the stirring process, centrifuge the suspension to eliminate any unbound NPs, and subsequently wash the composite with DI water for purification. The resulting MXene-Ag₂O NP composite typically remains in a solid physical state; depending on the specific application, the final composite may be dried under a vacuum or utilized directly in its solution form.

Anticancer activity

The MTT (3-(4,5-dimethylthiazol-2-yl)-2,5-diphenyl-2H-tetrazolium bromide) assay was used to measure cell viability and proliferation. In this colourimetric assay, colorectal cancer (CRC) cells (HCT116) are seeded into the wells of a multiwell plate. After adherence overnight, different concentrations of Ti₃C₂-Ag₂O nanosheets (very low to high concentration) are added to the cells. Control wells for nontreated cells (cells without treatment) and vehicle control (culture media only) are included. The cells are exposed to the nanosheets for 48 hours, which allows the cell-killing effect of the nanosheets. The MTT reagent is then added and incubated for another two to four hours in a CO₂ incubator, whereby metabolically active cells reduce the yellow tetrazolium salt, MTT, absorbed by each well to form purple MTT-formazan crystals. The culture media with MTT reagent is removed; dimethyl sulfoxide (DMSO) is added to dissolve the formazan crystals, giving rise to a purple solution.

## Results

XRD analysis

**Figure 1:**
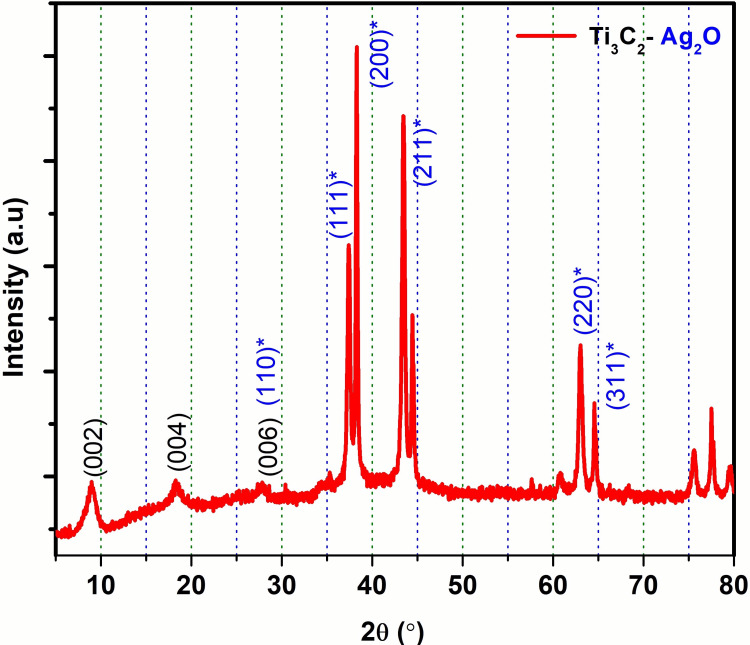
XRD analysis of Ti₃C₂-Ag₂O The asterisk (*) indicates that these peaks belong to the Ag₂O fraction of silver oxide in the composite XRD: X-ray diffraction; Ti₃C₂-Ag₂O: Titanium carbide (MXene)-Silver oxide

XRD patterns are used for the determination of crystal structure. The XRD patterns are essentially consistent with a composite of Ti₃C₂ (MXene) and Ag₂O, as shown in Figure 1. The horizontal axis represents the diffraction angle (2θ) in degrees. The peaks at typical 2θ values correspond to the planes of the crystal lattice where constructive interference occurs, indicating the existence of specific crystal planes. The vertical axis represents the intensity of diffracted X-rays; the number of scattered rays is parallel to the planes. High peaks indicate more prominent crystal planes. Peaks in bold correspond to Ti₃C₂, marked by indices such as (002), (004), and (006), which are typical for layered materials like MXenes. These peaks show a well-ordered, layered structure typical of MXenes. The position of the (002) peak is important because it can provide information about the interlayer spacing of the Ti₃C₂ material, which can vary depending on the surface chemistry and underlying species. The blue-labelled peaks, indicated by indices such as (111)* and (200)*, correspond to the Ag₂O phase. The asterisk (*) indicates that these peaks belong to the Ag₂O fraction of silver oxide in the composite. The sharpness and position of these peaks suggest a crystalline phase of Ag₂O. The presence of Ti₃C₂ and Ag₂O peaks in the XRD pattern confirms the presence of both phases in the composite. There is no evidence of a large peak displacement, indicating that the two components are independent of each other, whether fresh or in solid solution. The sharp peaks, especially those corresponding to Ag₂O, indicate that the Ag₂O in the composite is highly crystalline. Ti₃C₂ also exhibits good crystallinity, as indicated by its prominent layered surface. The XRD pattern does not show any other peaks that would indicate the formation of a new phase, such as a chemical reaction between Ti₃C₂ and Ag₂O to form a different mixture. Thus, the model shows that Ti₃C₂ and Ag₂O coexist as different phases. This XRD study indicates that the material is a composite of crystalline Ti₃C₂ and Ag₂O, with the two sides maintaining different crystal structures.

EDS analysis

**Figure 2:**
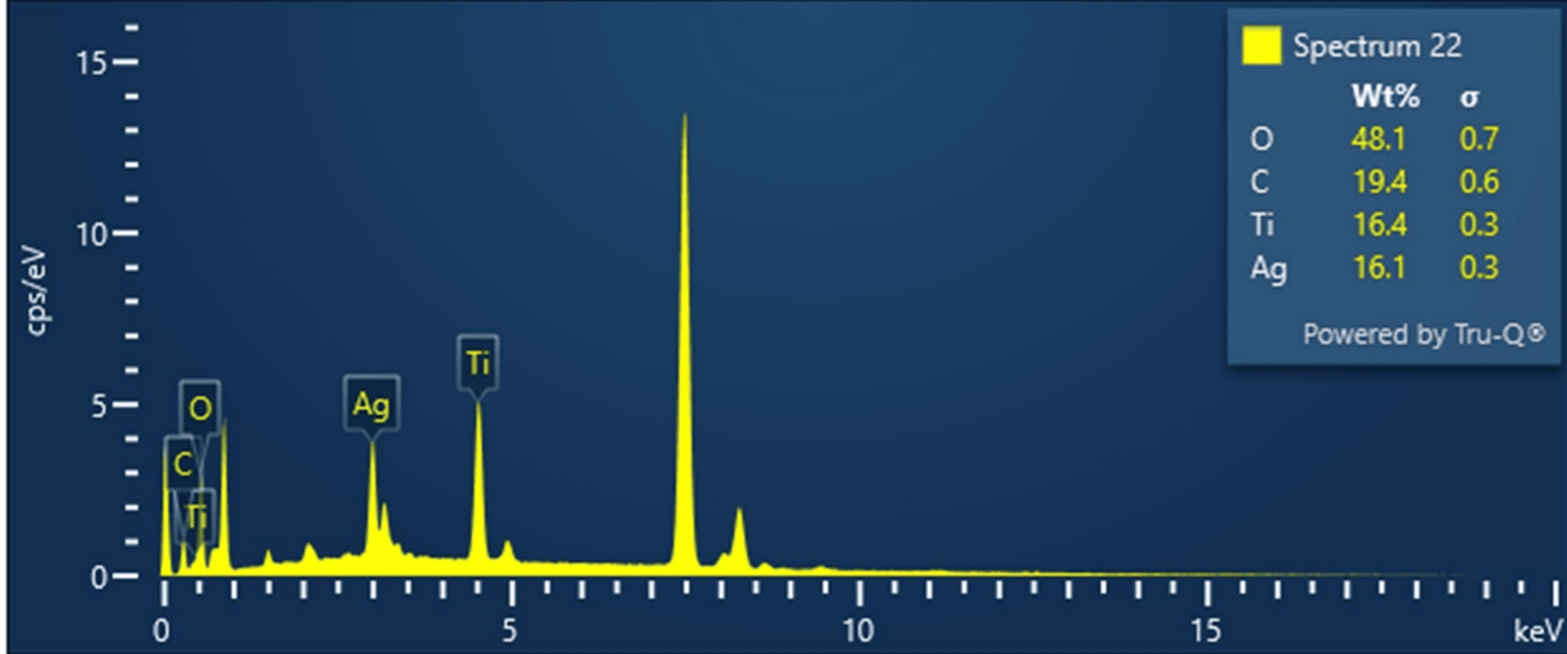
EDS analysis of Ti₃C₂-Ag₂O EDS: Energy dispersive spectroscopy; Ti₃C₂-Ag₂O: Titanium carbide (MXene)-Silver oxide

EDS, also known as energy dispersive X-ray spectroscopy (EDAX), is an X-ray technique used to characterize the elements in Ti₃C₂-Ag₂O samples. The Ti₃C₂ alloy consists mainly of titanium and carbon. The EDS spectrum reveals distinct X-ray peaks corresponding to elements in the sample, and the intensities of these peaks are used to quantify the relative abundance of each element. EDS mapping results (Figure 2) of the Ti₃C₂-Ag₂O compound show carbon (C), oxygen (O), titanium (Ti), and silver (Ag), indicating an even distribution of the four elements, with weight percentages of C, O, Ti, and Ag at 19.40%, 48.11%, 16.44%, and 16.05%, respectively, totalling 100%. This indicates the purity of the Ti₃C₂-Ag₂O alloy and confirms the absence of impurities, as shown in detail in Table 1.

**Table 1:** EDS analysis of Ti₃C₂-Ag₂O EDS: Energy dispersive spectroscopy; Ti₃C₂-Ag₂O: Titanium carbide (MXene)-Silver oxide

Element	Line type	Apparent concentration	k ratio	Wt%	Wt% sigma	Standard label	Factory standard
C	K series	0.51	0.00510	19.40%	0.64	C Vit	Yes
O	K series	1.50	0.00505	48.11%	0.68	SiO_2_	Yes
Ti	K series	1.55	0.01554	16.44%	0.27	Ti	Yes
Ag	L series	1.57	0.01572	16.05%	0.32	Ag	Yes
Total				100%			

SEM analysis

**Figure 3:**
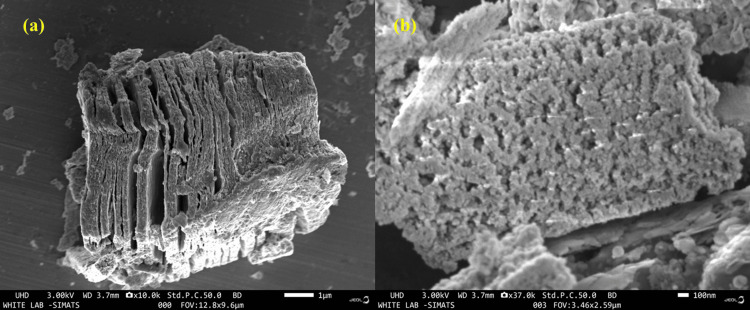
SEM analysis of Ti₃C₂-Ag₂O: a) 10k resolution and b) 37k resolution SEM: Scanning electron microscopy; Ti₃C₂-Ag₂O: Titanium carbide(MXene)-Silver oxide

SEM images of the Ti₃C₂ composite with Ag₂O NPs are shown. Figure 3a reveals the characteristic layered structure of Ti₃C₂, with stacked, sheet-like layers and distinct, sharp edges, indicating well-exfoliated MXenes. The surface appears smooth and free of visible NPs, suggesting that Ag₂O NPs may not yet be prominent in this region. Figure 3b provides a closer view, showing that the Ti₃C₂ surface is densely covered with small, spherical or granular particles, likely Ag₂O NPs. These particles are uniformly distributed, creating a rough, textured appearance on the MXene surface. The even coating of Ag₂O NPs confirms successful synthesis and effective adherence to the MXene. The composite exhibits a porous structure, potentially enhancing its surface area, which is advantageous for applications such as catalysis or drug delivery. Overall, the SEM images validate the successful formation of the Ti₃C₂ and Ag₂O NP composite, with the MXene retaining its layered structure and the Ag₂O NPs uniformly distributed.

Anticancer activity

**Figure 4:**
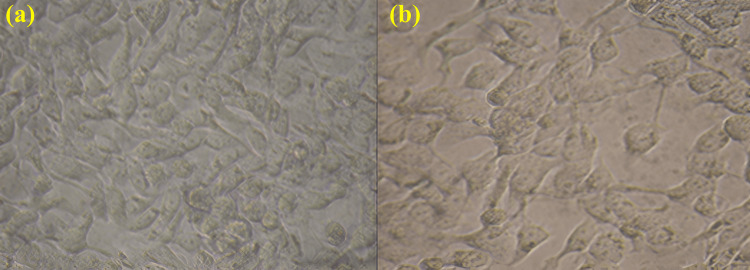
Anticancer activity of Ti₃C₂-Ag₂O: a) control and b) treatment Ti₃C₂-Ag₂O: Titanium carbide (MXene)-Silver oxide

The MTT assay is a colourimetric test used to evaluate cell metabolic activity and assess the cytotoxicity of potential anticancer compounds (Figure 4). In Figure 4a, cells are numerous and spread out, suggesting high viability and minimal impact from a lower concentration of the Ti₃C₂-Ag₂O composite. Conversely, Figure 4b shows fewer cells with signs of rounding or detachment, indicating reduced viability due to exposure to a higher concentration of the composite, leading to cytotoxic effects. The MTT assay visualizes cell viability by the purple formazan colour produced, with a more intense colour reflecting higher cell viability. Thus, the left panel indicates higher viability, while the right panel shows reduced viability. The decreased cell numbers or health in Figure 4b suggest that Ti₃C₂-Ag₂O exhibits cytotoxicity at higher concentrations. Cytotoxicity can be measured by comparing absorbance readings from the assay, with higher absorbance indicating better cell viability. The results imply that the Ti₃C₂-Ag₂O composite has anticancer potential, with its activity quantified through a dose response. Higher concentrations appear to induce cell death or inhibit growth, suggesting Ti₃C₂-Ag₂O exhibits potential as an anticancer agent.

## Discussion

MXenes are recognized as highly promising materials due to their significant surface area, outstanding thermal and electrical conductivity, and tunable band gap. Additionally, they exhibit exceptional hydrophilicity and stability. The adsorption and reduction properties of MXene-based 2D nanomaterials make them efficient photocatalysts for degrading organic pollutants [12]. XRD patterns of different molar ratios of Ag₂O, pure TiO₂, and Ag₂O/TiO₂ nano heterojunction composites reveal different characteristics: 2θ = 32.8°, 38.1°, 54.9°, 65.4°, and 68.8° for Ag₂O. Here, peaks {111}, {200}, {220}, {311}, and {222} correspond to the crystal planes, as described in the Joint Committee on Powder Diffraction (JCPDS) 41-1104. In pure TiO₂, the peaks at 2θ = 25.27°, 37.80°, 47.92°, 53.93°, 54.94°, 62.67°, 68.79°, 70.16°, and 75.01° are located on the anatase crystal faces {101}, {103}, {004}, {200}, {200}, {105}, {211}, {204}, {116}, {220}, and {215}, according to JCPDS 12-1272 [13]. All observed peaks for the Ag₂O/TiO₂ nano heterostructure (curve c) correspond to either TiO₂ or Ag₂O, with no other impurity peaks observed. The absence of a distinct Ag₂O peak indicates that Ag₂O has a relatively low concentration, suggesting that TiO₂ does not form new network bonds with Ag₂O NPs on the TiO₂ surface {101}. The growth of the plane is inhibited [14].

EDS analyses of the Ti₃C₂/Al₂O₃/Ag, Ti₃C₂/SiO₂/Ag, and Ti₃C₂/SiO₂/Pd nanocomposites reveal the presence of signals corresponding to C, Ti, O, and F, which are uniformly detected across all samples. Additionally, signals indicative of Ag confirm the presence of silver in the Ti₃C₂/Al₂O₃/Ag and Ti₃C₂/SiO₂/Ag samples [15]. EDS is an X-ray technique used to analyse the properties of Ti₃C₂-BiOCl samples. The Ti₃C₂ structure consists mainly of titanium and carbon. The EDS spectrum reveals specific X-ray peaks corresponding to elements in the sample, and their intensities are used to quantify each element. EDS map results include carbon (C), oxygen (O), titanium (Ti), bismuth (Bi), and chloride (Cl), showing uniform distribution. The weight percentages of C, O, Ti, Bi, and Cl were determined to be 32.45%, 27.12%, 25.64%, 14.79%, and 0.00%, respectively, confirming that the product is pure and free of impurities. Furthermore, EDS analysis verifies the absence of other substances, indicating that Ti₃C₂-BiOCl is free of impurities [16]. The morphology of Ti₃C₂-MXene, Ti₃C₂/Al₂O₃/Ag, Ti₃C₂/SiO₂/Ag, and Ti₃C₂/SiO₂/Pd nanocomposites was analysed by SEM. The SEM images clearly show the characteristic structure of Ti₃C₂ MXene, which is explained by nano-sized sheets where crack-like holes are present. The Ti₃C₂ structure underwent surface modification by incorporation of composite NPs, mainly Al₂O₃/Ag, SiO₂/Ag, and SiO₂/Pd. Moreover, SEM analysis showed that the composite NPs exhibit different sizes, mainly still less than 100 nm, which verifies the successful development of the nanocomposite systems [15]. The anticancer properties of Ti₃C₂-BiVO₄ nanosheets have been demonstrated by cell death studies, especially MTT studies using colon cancer cell lines, which revealed significant anticancer activity. Ti₃C₂-BiVO₄ nanosheets exhibit photocatalytic properties that generate reactive oxygen species (ROS) upon exposure to light [17]. These ROS, including singlet oxygen (~¹O₂) and superoxide radicals (O₂⁻), induce DNA damage, lipid peroxidation, and protein oxidation in cancer cells, leading to oxidative stress, followed by apoptotic cell death. Small nanosheets and their unique properties facilitate their uptake and localization in the cytoplasm and nucleus of cancer cells, enhancing their anticancer potential. The addition of BiVO₄ improves the biocompatibility and stability of the nanosheets, resulting in effective treatment [18]. Ti₃C₂-BiVO₄ nanosheets impair mitochondrial function in CRC cells by decreasing mitochondrial membrane depolarization and adenosine triphosphate (ATP) production, while increasing the release of cytochrome c into the cytoplasm, leading to cell death, DNA fragmentation, and activation of the apoptotic pathway. Moreover, treatment with these nanosheets induces cell cycle arrest by upregulating cell cycle inhibitors such as p21 and p27, and downregulating cyclins and cyclin-dependent kinases (CDKs) in the G0/G1 or G2/M phases. This disorder inhibits cell cycle progression and induces apoptotic cell death [19]. Ti₃C₂-BiVO₄ nanosheets also affect key signalling pathways of CRC progression, including PI3K/AKT/mTOR, MAPK/ERK, and Wnt/β-catenin, leading to decreased CRC cell motility, invasion, and survival. Furthermore, the immunomodulatory effects of these nanosheets enhance the immune response to CRC by activating cytotoxic T-lymphocytes (CTLs), natural killer (NK) cells, and dendritic cells (DCs) in the tumour microenvironment, helping to identify and disrupt the tumour microenvironment in cancer cells. Overall, Ti₃C₂-BiVO₄ nanosheets exhibit various anticancer activities, such as induction of ROS-mediated oxidative stress, mitochondrial dysfunction, cell cycle arrest, inhibition of important signalling pathways, and enhanced immune response, emphasizing their potential as a treatment for CRC [20].

Limitation

The optimization of Ti₃C₂-Ag₂O nanostructures for targeted cancer therapy faces several limitations. Scaling up synthesis methods from laboratory to industrial production may be challenging and could impact quality or efficiency. Long-term stability in biological environments requires thorough investigation, as NPs may degrade or alter properties over time. Achieving uniform and effective functionalization is crucial, as incomplete functionalization can lead to inconsistent performance. In vitro, anticancer activity must be validated, with further assessment of cytotoxicity and biocompatibility in vivo to ensure safety. The effectiveness of targeted delivery needs optimization to ensure selective accumulation in cancer tissues without affecting healthy cells. Interactions with biological systems, including potential immune responses, must be studied in detail. Additionally, navigating regulatory and safety concerns through comprehensive preclinical and clinical trials is essential, and the cost-effectiveness of production needs evaluation to ensure feasibility for clinical use.

## Conclusions

Enhancement of Ti₃C₂-Ag₂O nanostructures for targeted cancer therapy demonstrates significant potential for advancing therapeutic strategies. The synthesis process effectively integrates Ti₃C₂ MXenes with Ag₂O NPs, resulting in a composite with enhanced properties suited for targeted applications. Functionalization techniques, including surfactants and chemical reduction processes, further improve the stability and efficacy of the nanostructures. In vitro evaluations reveal that the Ti₃C₂-Ag₂O composite exhibits promising anticancer activity, with dose-dependent cytotoxic effects observed in cancer cell lines. The successful incorporation of Ag₂O NPs onto Ti₃C₂ surfaces, and their uniform distribution, contribute to the composite’s high surface area and interaction with cancer cells. These findings underscore the composite’s potential as an effective and targeted therapeutic agent, warranting further investigation and optimization to enhance its clinical applicability and performance in cancer treatment.
